# A Rapid Field-Visualization Detection Platform for Genetically Modified Soybean ‘Zhonghuang 6106’ Based on RPA-CRISPR

**DOI:** 10.3390/ijms26010108

**Published:** 2024-12-26

**Authors:** Ran Tao, Jihong Zhang, Lixia Meng, Jin Gao, Chaohua Miao, Weixiao Liu, Wujun Jin, Yusong Wan

**Affiliations:** 1 Biotechnology Research Institute, Chinese Academy of Agricultural Sciences, Beijing 100081, China; taoran8833@163.com (R.T.); zhangjihong@caas.cn (J.Z.); menglixia2024@163.com (L.M.); gaojinflying@163.com (J.G.); miaochaohua@caas.cn (C.M.); liuweixiao@caas.cn (W.L.); 2 Inspection and Testing Center for Ecological and Environmental Risk Assessment of Plant and Plant-Related Microorganisms (Beijing), Ministry of Agriculture and Rural Affairs, Beijing 100081, China

**Keywords:** CRISPR-Cas12a, recombinase polymerase amplification, GM soybean, nucleic acid detection, point-of-care testing

## Abstract

Genetically modified (GM) herbicide-tolerant soybean ‘Zhonghuang 6106’, which introduces a glyphosate-resistant gene, ensures soybean yield while allowing farmers to reduce the use of other herbicides, thereby reducing weed management costs. To protect consumer rights and facilitate government supervision, we have established a simple and rapid on-site nucleic acid detection method for GM soybean ‘Zhonghuang 6106’. This method leverages the isothermal amplification characteristics of RPA technology and the high specificity of CRISPR-Cas12a to achieve high sensitivity and accuracy in detecting GM soybean components. By optimizing experimental conditions, the platform can quickly produce visual detection results, significantly reducing detection time and improving efficiency. The system can detect down to 10 copies/μL of ‘Zhonghuang 6106’ DNA templates, and the entire detection process takes about 1 h. The technology also has strong editing capabilities; by redesigning the primers and crRNA in the method, it can become a specific detection method for other GM samples, providing strong technical support for the regulation and safety evaluation of GM crops.

## 1. Introduction

With the rapid advancement of biotechnology, GM crops have gradually become an important part of modern agriculture. These crops, through genetic modification, can significantly enhance crop yield, resistance to stress, and nutritional value, playing an important role in agricultural production [1]. In China, soybeans, a key oil and protein crop, cover a planting area of 10.47 million hectares, with an annual production of 20.84 million tons. To accelerate the pace of biotechnological breeding, several high-quality soybean varieties have been independently developed. Among them, the GM soybean ‘Zhonghuang 6106’ is not only herbicide-resistant but also offers yield advantages, showing great potential for widespread application. However, as GM crops become more prevalent, consumer concerns about food safety continue to increase, and so does the demand for the detection of GM content. This is necessary not only to ensure food safety but also to comply with regulatory requirements [2]. As a result, the development of precise and rapid GM detection technologies has become a key need in food safety and agricultural regulation.

Currently, traditional nucleic acid detection technologies, such as polymerase chain reaction (PCR) and quantitative real-time PCR (qPCR), are widely used for detecting GM content. These methods, known for their high sensitivity and specificity, can accurately identify target nucleic acid sequences in laboratory settings and have become standard techniques in molecular biology [3,4]. PCR amplifies specific DNA fragments through thermal cycling [5], thereby achieving efficient detection of target sequences, while qPCR allows real-time monitoring of the amplification process, providing even more accurate detection [6,7]. Although PCR and qPCR technologies perform well under laboratory conditions, they also have clear limitations. These methods rely on complex instrumentation, such as thermal cyclers and fluorescence-based quantitative detection systems, which are not only expensive but also require trained personnel for operation [8]. The traditional PCR detection process usually takes a long time, with sample processing that can take several hours to days to result in an output, making it unsuitable for rapid on-site detection [9].

To address these challenges, scientists have been exploring more convenient and efficient nucleic acid detection methods to meet the demand for rapid field detection. Recombinase polymerase amplification (RPA) technology has emerged as an important amplification technique in the field of nucleic acid detection due to its advantages, including no need for thermal cycling equipment, simple operation, and high detection sensitivity. RPA is an isothermal amplification technique that amplifies target fragments through recombinase-mediated binding of primers to the template DNA, followed by polymerase extension [10,11]. Compared to PCR, RPA operates under more relaxed conditions, allowing amplification at lower temperatures (typically between 37 °C and 42 °C), with significantly shorter reaction times, usually completed within 20 to 30 min [12]. This makes RPA particularly suitable for on-site detection and use in resource-limited environments. Additionally, RPA requires minimal equipment, making nucleic acid amplification possible with portable or handheld devices, greatly expanding its application scenarios [13]. With these advantages, RPA technology has been widely used in areas such as disease diagnosis, food safety testing, and environmental monitoring, becoming a common nucleic acid detection method.

**Figure 1:**
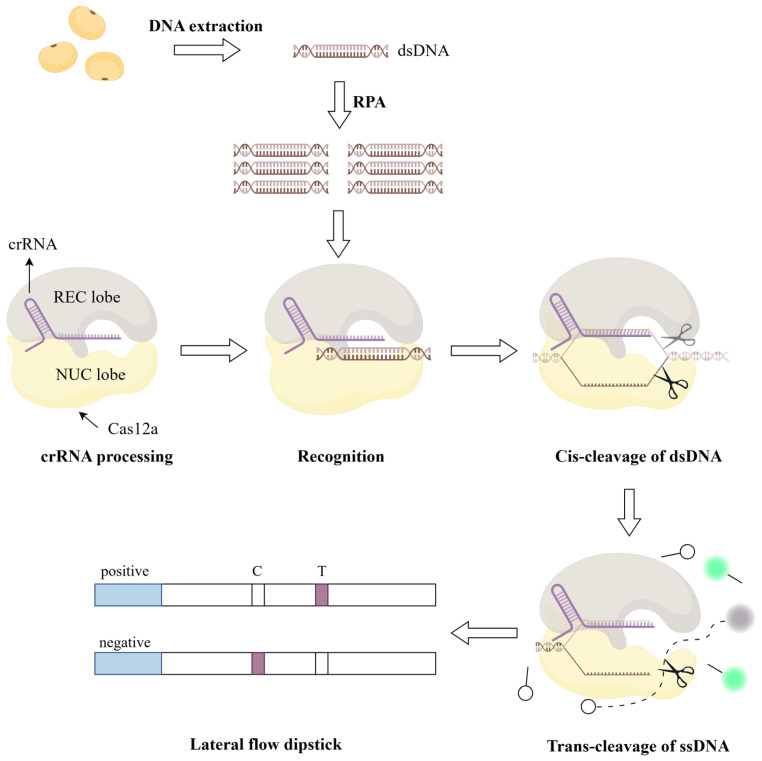
Detection principle of the RPA-CRISPR method. Genomic DNA is extracted from soybean seeds or buds, and the target fragment is rapidly amplified using RPA technology. The RNP complex composed of crRNA and Cas12a protein can precisely identify the target and cleave double-stranded DNA, known as cis-cleavage. Subsequently, the Cas12a protein remains activated and non-specifically cleaves single-stranded DNA within the system, known as trans-cleavage. By adding single-stranded fluorescent signal molecules to the system and combining with lateral flow dipsticks, rapid visual detection can be achieved. Figure created using Figdraw (version 2.0).

CRISPR technology is a gene-editing tool based on the bacterial immune system that has recently attracted widespread attention in the field of molecular biology [14,15]. CRISPR technology relies on Cas proteins and crRNA to precisely identify and cleave target nucleic acid sequences, exhibiting high specificity and flexibility. By combining CRISPR with RPA technology, rapid and efficient on-site detection can be achieved while amplifying the target nucleic acid sequences [16]. Specifically, RPA technology first amplifies the target gene fragments, and then the CRISPR system, composed of Cas proteins and crRNA, precisely identifies the amplified products, activates trans-cleavage activity, and combines with visual detection methods such as lateral flow dipsticks to achieve rapid result output (Figure 1). This technological combination greatly enhances detection sensitivity and specificity while simplifying the operational process [17,18,19].

The combination of RPA-CRISPR technology has demonstrated significant advantages in the detection of GM crops, especially in fast and convenient on-site testing [20]. RPA achieves isothermal amplification of the target sequence at low temperatures, eliminating the need for traditional thermal cycles, while CRISPR relies on its highly specific gene recognition capability to precisely distinguish target sequences in genetically modified crops [21]. This technological integration not only surpasses individual methods in terms of sensitivity but is also simple to operate and requires minimal equipment, making it ideal for detection in resource-limited or field conditions [22]. Furthermore, RPA-CRISPR technology allows for the detection results to be visually interpreted, further improving its convenience for on-site operations [23].

This study aims to develop a detection method for GM soybean ‘Zhonghuang 6106’ based on RPA-CRISPR technology. By designing primers and probes based on the event-specific sequence and combining the rapid amplification capability of RPA with the high specificity of CRISPR-Cas12a recognition, we expect to establish a platform for the rapid detection of genetically modified components in the field. The successful development of this method will provide strong technical support for on-site detection of GM soybeans and is of significant importance for ensuring the quality of agricultural products and promoting the application of biotechnology in agriculture.

## 2. Results

### 2.1. Establishment and Optimization of RPA Method for ‘Zhonghuang 6106’

**Figure 2:**
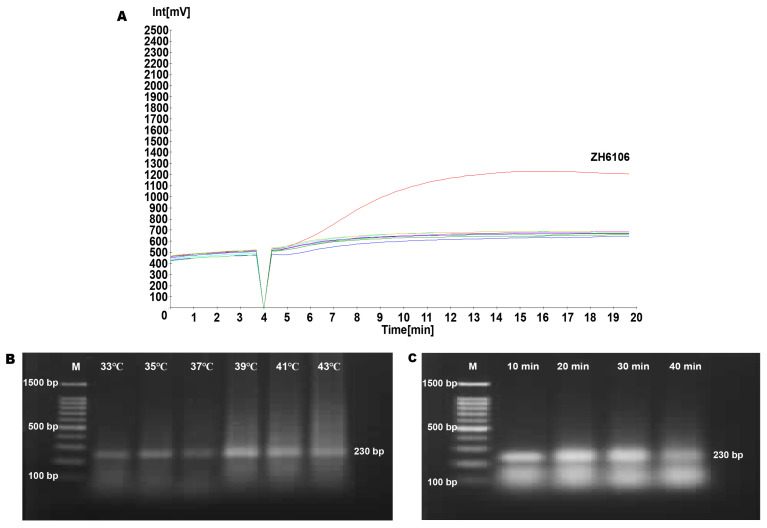
RPA primer screening and condition optimization. (**A**) RPA primer specificity fluorescence experiment. The red curve represents genetically modified soybean ‘Zhonghuang 6106’, and the other curves are mixed samples of various genetically modified crops and blank controls. (**B**) RPA reaction temperature optimization. M represents the 100 bp DNA ladder, with the smallest fragment being 100 bp and the largest fragment being 1500 bp; the target fragment size in the experimental group is 230 bp. (**C**) RPA reaction time optimization. The bands at 20 min and 30 min are significantly brighter than the other two bands.

To establish the RPA detection method for ‘Zhonghuang 6106,’ we designed 80 primer combinations and systematically screened them, observing the formation of target bands by agarose gel electrophoresis. The results are provided in Supplementary Figure S1. Specificity tests for the selected primers were conducted on various samples, including ‘Zhonghuang 6106’, ‘Zhonghuang 10’ and mixed samples of several GM crops, such as GM maize, GM rice, GM cotton, GM rapeseed, and GM soybeans that did not contain ‘Zhonghuang 6106’. The fluorescence detection results indicated that a significant fluorescence signal was only detected when using ‘Zhonghuang 6106’ DNA as a template, while no fluorescence signals were observed in other samples (Figure 2A). This demonstrates the good specificity of the screened primers.

Next, we optimized the temperature and time for the RPA reaction. Temperature optimization experiments were performed at five different temperatures (33 °C, 35 °C, 37 °C, 39 °C, 41 °C, and 43 °C). The results showed that the brightness of the observed bands was most significant at 39 °C; when the temperature exceeded 39 °C, the brightness of the bands was reduced. Therefore, 39 °C was determined to be the optimal temperature for the RPA reaction (Figure 2B).

Using a DNA template concentration of 0.25 ng/μL, we further optimized the reaction time at four different intervals (10 min, 20 min, 30 min, and 40 min). The electrophoresis results (Figure 2C) indicated that target fragments could be amplified after just 10 min, while the brightness of the bands for 20 min and 30 min was similar, with the band brightness at 40 min being the weakest. Considering the impact of reaction time on detection limits and the efficiency of the overall detection process, we ultimately selected 20 min as the optimal time for the RPA reaction.

### 2.2. Feasibility of the RPA-CRISPR Method

**Figure 3:**
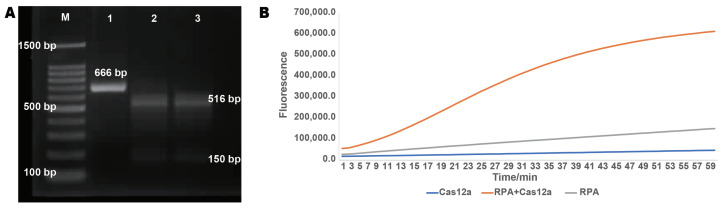
Feasibility analysis of the RPA-CRISPR method. (**A**) crRNA validation experiment. Lane 1 is the negative control without crRNA, with a fragment size of 666 bp. Lanes 2 and 3 are two parallel experimental groups with crRNA added, with fragment sizes of 516 bp and 150 bp, respectively. (**B**) Compatibility test of RPA reaction products and CRISPR system. Fluorescence signals were observed only when both Cas12a protein and RPA products were present in the system.

To validate the feasibility of the RPA-CRISPR method, it is essential first to confirm the effectiveness of the crRNA. For this purpose, we amplified a 666 bp fragment by PCR that encompasses the target site. Theoretically, the Cas12a protein should cleave the PCR fragment at this target, generating two fragments of 150 bp and 516 bp. In the experiment, we incubated the crRNA with the Cas12a protein at 37 °C to form a complex, followed by the addition of 250 ng of PCR product and a 1 h reaction at 37 °C. The results were then analyzed using agarose gel electrophoresis (Figure 3A). Electrophoresis analysis revealed that the PCR fragments in the experimental group were completely digested by the Cas12a protein compared to the control group, indicating that the designed crRNA effectively recognized the target and guided the Cas12a protein for cleavage.

Next, to assess the compatibility of the RPA amplification system with the CRISPR-Cas12a system, we designed three experimental groups and included a probe to detect fluorescence signal intensity using the QuantStudioTM 3 Real-Time PCR instrument. In experimental group one, both the RPA product and the Cas12a protein were added simultaneously; experimental group two included only the Cas12a protein; and experimental group three contained only the RPA product. The real-time fluorescence detection results (Figure 3B) showed that fluorescence signals were observed only when both the RPA product and the CRISPR-Cas12a system were present, indicating that the trans-cleavage reaction could only occur under their combined effect. Overall, these experimental results fully validate the feasibility of the RPA-CRISPR-Cas12a method for detecting ‘Zhonghuang 6106’.

### 2.3. Optimization of the RPA-CRISPR Reaction System Conditions

**Figure 4:**
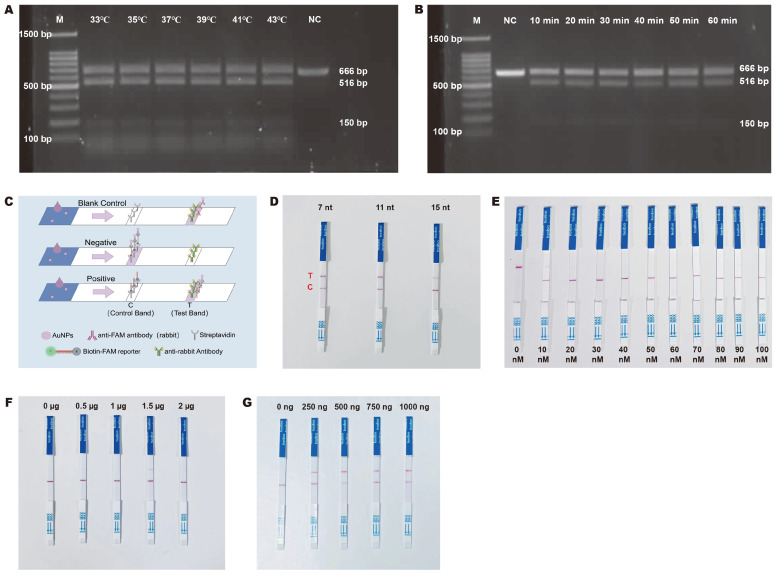
Optimization of the RPA-CRISPR Reaction System. (**A**) CRISPR reaction temperature optimization. The experimental group contained Cas12a protein, which hindered the movement of DNA fragments in agarose during electrophoresis, causing them to move more slowly. In fact, the size of the top fragment in the experimental group was consistent with that of the negative control group. (**B**) CRISPR reaction time optimization. The reaction time was determined based on the brightness of the band at the 516 bp position; the bands at 30 min and 50 min were relatively bright, while the bands at 40 min and 60 min were the darkest. (**C**) Principle of the lateral flow dipstick. For positive samples, the AuNPs–anti-FAM antibody complexes bind with the anti-rabbit antibody of the test band and generate purple signal. On the contrary, for negative samples, the reporter is not cut and forms a new complex with the AuNPs–anti-FAM antibody complex that binds the control band site. Figure created using Figdraw. (**D**) Reporter length selection. T represents the test line (test), and C represents the control line (control). The T line color of the 11 nt reporter was the deepest. (**E**) Reporter screening. The experimental groups at 10 nM-30 nM all had a faint color at the T line, and at 40 nM, there was no color at the T line. (**F**) Cas12a protein screening. When the protein amount was 1.5 ng, the T line color was the deepest. (**G**) crRNA screening. The color of the T line on the test strip deepened with the increase in the amount of crRNA.

To achieve better visual detection results with the RPA-CRISPR-Cas12a method, we systematically optimized the reaction temperature and time and selected the reporter while investigating the optimal amounts of Cas12a, crRNA, and reporter. During the determination of the optimal reaction time and temperature, we employed agarose gel electrophoresis to assess the best conditions by observing the brightness of the bands. The electrophoresis results indicated that there was no significant difference in band brightness within the temperature range of 33 °C to 43 °C, suggesting that temperature had a minimal effect on the reaction in this interval (Figure 4A). In the exploration of reaction time (Figure 4B), the bands at the 516 bp position were notably bright at both 30 min and 50 min. Considering the overall time efficiency of the detection process, we ultimately selected 30 min as the optimal time for the CRISPR reaction.

According to the working principle of the lateral flow dipstick (Figure 4C), AuNPs (Gold Nanoparticles) first form a complex with anti-FAM antibodies (rabbit). When there is no reporter in the system, the complex flows to the farthest end of the test strip, i.e., the test line (T line); when the reporter is present, the AuNPs–anti-FAM antibody complexes bind with the FAM group on the reporter, forming a new complex, which is captured by Streptavidin on the test strip and stays at the control line (C line). However, when the amount of reporter is insufficient, it cannot bind with all AuNPs–anti-FAM antibody complexes, and some complexes flow to the T line, causing false-positive results. Therefore, in the experiment, the lowest concentration at which the C line appears but the T line does not is the optimal concentration for the reporter. Next, we explored the optimal length and amount of the reporter; designed reporters with lengths of 7 nt, 11 nt, and 15 nt (Supplementary Table S1); and set up a concentration gradient from 0 nM to 100 nM. According to the color development of the test strip (Figure 4D), the 11 nt reporter had the deepest color and the shortest color development time, so we chose the 11 nt reporter. Experimental results showed that 40 nM was the optimal concentration for the reporter (Figure 4E). In subsequent experiments (Figure 4F,G), we determined the optimal amounts of Cas12a protein and crRNA to be 1.5 μg and 1000 ng, respectively, and the results obtained by the real-time fluorescence method were consistent with those of the colloidal gold method (Supplementary Figure S2A,B). Finally, within a 30 min reaction time, the reaction system contained 0.348 μM Cas12a protein, 36.2 μM crRNA, and 0.4 μM reporter.

### 2.4. Specificity of the RPA-CRISPR Method

**Figure 5:**
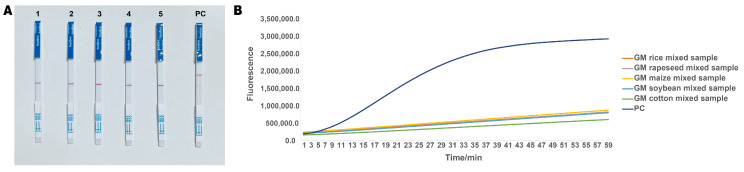
Specificity of the RPA-CRISPR method. (**A**) Results from the lateral flow dipsticks: 1. GM rice mixed sample; 2. GM rapeseed mixed sample; 3. GM maize mixed sample; 4. GM soybean mixed sample; 5. GM cotton mixed sample; PC: positive control. (**B**) Real-time fluorescence results, consistent with the lateral flow dipstick results.

To assess the specificity of the RPA-CRISPR assay for detecting ‘Zhonghuang 6106’, we designed experiments to test ‘Zhonghuang 6106’ samples alongside mixed samples of other transgenic crops. In this experiment, ‘Zhonghuang 6106’ DNA was used as the positive control, and the results demonstrated that significant positive bands were observed only in the positive control, while no bands were detected in the mixed samples of other GM crops (Figure 5A). Additionally, the specificity was further validated using real-time fluorescence detection (Figure 5B), which showed that the fluorescence signals were consistent with the results from the lateral flow dipsticks. Based on these experimental data, we concluded that the established RPA-CRISPR assay exhibits high specificity in detecting ‘Zhonghuang 6106’ and can accurately identify ‘Zhonghuang 6106’ samples without interference from other GM crop samples.

### 2.5. LOD of the RPA-CRISPR Method

**Figure 6:**
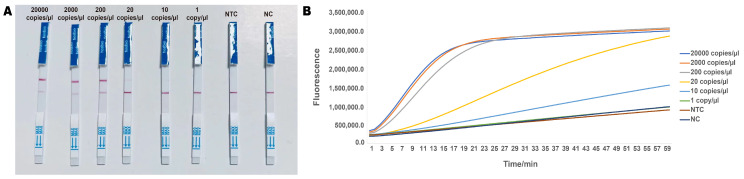
LOD of the RPA-CRISPR method. (**A**) Lateral flow test strip results. A faint band was observed at 10 copies/μL. NTC, no template control. (**B**) Real-time fluorescence results. The LOD was 10 copies/μL. The addition of RPA products to the CRISPR system caused partial degradation of the reporter, leading to a weak signal in the 1 copy/μL group, NTC, and NC, but this did not affect the lateral flow test strip results.

To determine the limit of detection (LOD) of the RPA-CRISPR method for ‘Zhonghuang 6106’, we tested samples with varying DNA template copies of ‘Zhonghuang 6106’ (Figure 6A). When the copies ranged between 200 copies/μL and 20,000 copies/μL, the bands on the test strips were clear and appeared quickly. Even for the groups with 10 copies/μL to 20 copies/μL, the bands, though lighter in color, were still distinguishable. The color development time was kept within 10 min, meeting the requirement for rapid detection. Subsequently, we further validated the LOD using real-time fluorescence (Figure 6B), and the results were consistent with those from the lateral flow test strips. These findings indicate that the established RPA-CRISPR method demonstrates high sensitivity, with a detection limit of 10 copies/μL.

### 2.6. Application of TianGen Rapid DNA Extraction Kit

**Figure 7:**
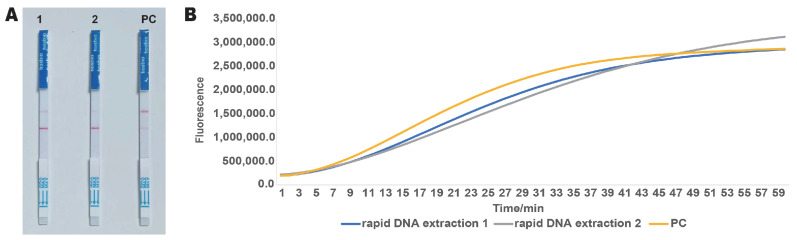
Application of the rapid DNA extraction method. (**A**) Lateral flow test strip results. 1 and 2 represent two parallel test groups. PC represents positive control. (**B**) Real-time fluorescence results. At the 30 min mark, the fluorescence signal intensity in the test group was weaker than that in the positive control, consistent with the lateral flow test strip results.

To improve detection efficiency and reduce DNA extraction time, we utilized the TianGen Rapid DNA Extraction Kit to extract genomic DNA from ‘Zhonghuang 6106’ and compared it with DNA templates extracted using a conventional plant genomic extraction kit. Based on the results from the lateral flow test strips (Figure 7A), the band color in the rapid extraction group was slightly lighter than that of the positive control, but the positive result was still clearly discernible. Real-time fluorescence detection results (Figure 7B) indicated that the fluorescence signal intensity of the rapid extraction group was lower than that of the control group before 47 min, but after that, the signal intensity of both groups became consistent, which aligned with the lateral flow test strip results. These findings demonstrate that the DNA template obtained using the rapid DNA extraction kit is suitable for the RPA-CRISPR detection of ‘Zhonghuang 6106’. Furthermore, this method significantly enhances extraction efficiency compared to conventional DNA extraction methods.

### 2.7. Application of the RPA-CRISPR Method for Zhonghuang 6106 Detection

**Figure 8:**
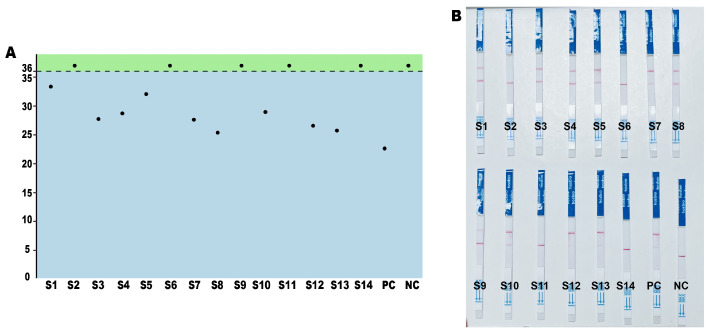
Application of the RPA-CRISPR method in real sample detection. (**A**) Ct values from qPCR analysis of 14 samples. (**B**) Lateral flow test strip results.

To validate the practical application of the RPA-CRISPR detection platform in real-world field testing, we conducted blind tests on soybean samples, including nine positive samples of ‘Zhonghuang 6106’ and five negative samples of ‘Zhonghuang 10’. First, the soybean samples were confirmed using real-time quantitative PCR (qPCR), followed by verification of the accuracy of the method using lateral flow test strips. Based on the Ct values obtained from qPCR and following the guidelines from the ‘Detection of genetically modified plants and derived products event-specific PCR methods for herbicide-tolerant soybean zhonghuang6106’ (draft), samples with Ct values greater than 36 were determined as negative. Samples S2, S6, S9, S11, and S14 were confirmed as negative (Figure 8A). The lateral flow test strip results showed distinct band colors for all positive samples, with sample S9 showing a faint positive band, while the remaining negative samples showed no bands (Figure 8B). In summary, the accuracy of the RPA-CRISPR method was 92.8%, and its sensitivity was 100%. This method can be effectively applied to the on-site detection of GM soybean ‘Zhonghuang 6106’.

## 3. Discussion

Over the past few decades, with the increasing growth of global agricultural trade, the cultivation and trade of GM crops have also expanded rapidly. However, the transnational movement of GM crops has introduced challenges in detection and monitoring, particularly in determining whether GM crops comply with the import and export standards of various countries [1]. GM soybean ’Zhonghuang 6106’, as the second soybean variety in China to obtain a biosafety certificate, requires rapid and accurate on-site detection to ensure agricultural safety, environmental protection, and consumer health. Traditional GM crop detection methods, such as PCR technology, although highly accurate, are complex, time-consuming, and heavily reliant on specialized equipment, which limits their applicability in on-site testing. To overcome these limitations, the combined use of RPA and CRISPR technologies has emerged as a promising new research direction, demonstrating substantial potential for low equipment requirements and high-sensitivity detection [24].

Huang et al. [25] proposed a multi-temperature real-time fluorescence quantitative PCR system for detecting human cytomegalovirus (HCMV), which can complete detection in a very short time; however, the experimental system is more complex than conventional qPCR methods. To improve detection sensitivity, Feng et al. [26] developed a droplet digital PCR method. However, both of these methods require high-end equipment, posing significant challenges for meeting the demands of on-site detection. In contrast, the advantage of the method presented in this study lies in the complementary integration of RPA and CRISPR technologies. RPA, a low-temperature amplification technique, enables stable reactions at lower temperatures (37–42 °C) and can be operated under various conditions, such as with warm water, room temperature, or even body temperature. CRISPR-Cas12a, on the other hand, provides high specificity and sensitivity for nucleic acid cleavage, allowing the entire detection system to deliver rapid and accurate visual results under field conditions [10].

The visual RPA-CRISPR detection platform developed in this study fully considers the practical requirements of on-site testing, offering significant advantages such as ease of operation, rapid reaction time, high sensitivity, and low equipment requirements. Within 50 min, the reaction can be completed using a simple heating device, and the results can be observed with the naked eye. The detection limit of this assay is 10 copies/μL. This greatly enhances detection efficiency and simplifies the complex steps typically required in traditional PCR testing. Compared to conventional laboratory methods, this platform not only significantly shortens the detection time but also reduces dependence on high-end equipment, making on-site detection more convenient. Additionally, when used with a rapid DNA extraction kit, the entire process—from DNA extraction to final result—takes only about 1 h, which provides a clear advantage for field applications. Zhu et al. reported a rapid detection method for GM papaya based on RPA-CRISPR-Cas12a, which utilizes UV excitation and was able to detect DNA templates at 20 copies [19]. Therefore, the visual detection method outlined in this study can be used for rapid and accurate GM soybean screening at entry and exit points, agricultural sites, and other locations.

Nevertheless, we acknowledge certain limitations in this study. While the potential of the RPA-CRISPR method for on-site environments has been demonstrated, a major challenge for future development, particularly in the context of high-throughput detection and large-scale applications, will be improving the automation of the detection platform to reduce human error and enhance batch processing capacity. Overall, the RPA-CRISPR-based GM soybean ’Zhonghuang 6106’ on-site visual detection platform developed in this study provides a simple, efficient, and cost-effective solution for rapid on-site detection of GM crops. This platform not only improves the efficiency and accuracy of on-site detection but also holds promise as a valuable reference for the detection of other GM crops. Future developments could consider applying this platform for the detection and development of other GM crops, such as herbicide-tolerant rice [27] and maize [28], to provide more diverse detection solutions for different types of GM crops. With further technological advancements and optimization, it is expected that this detection method will play an increasingly important role in agricultural safety, food monitoring, and the quarantine of GM crops in the future.

## 4. Materials and Methods

### 4.1. Materials and Reagents

The non-GM soybean ‘Zhonghuang 10’ and GM soybean ‘Zhonghuang 6106’ used in this experiment were provided by the Institute of Crop Science, Chinese Academy of Agricultural Sciences. The Twist Amp Basic Kit was purchased from TwistDX Ltd. (Maidenhead, UK). Primers, probes, and DEPC-treated water used in this experiment were synthesized or purchased from Sangon Bioengineering Co., Ltd. (Shanghai, China). Plant genomic DNA extraction kits and DNA rapid extraction kits were purchased from TianGen Biochemical Technology Co., Ltd. (Beijing, China). Ni-NTA Agarose and MinElute PCR Purification Kit were purchased from QIAGEN.N.V (Shanghai, China). Trans 10 and BL21 competent cells were purchased from Transgen Biological Technology Co., Ltd. (Beijing, China). NEBuffer 3 (10×) and HiScribe^®^ T7 High-Efficiency RNA Synthesis Kit were purchased from New England Biolabs (Beijing, China). Phanta Mix (6×) was purchased from Nanjing Vazyme Biotech Co., Ltd. (Nanjing, China). Premix Ex Taq (probe qPCR) and ROX Reference Dye II were purchased from Takara Technology Co., Ltd. (Beijing, China).

### 4.2. Nucleic Acid Extraction and Purification

The soybean seeds were ground into powder using a Mixer Mill MM 400 (Retsch, Haan, Germany). Genomic DNA was then extracted from the soybean powder using the TianGen Plant Genomic DNA Extraction Kit (TIANGEN, Beijing, China). The DNA concentration was determined using a NanoDrop 1000 UV spectrophotometer (Thermo Fisher Scientific, Waltham, MA, USA), and the DNA was diluted to 25 ng/μL and stored at −20 °C for further analysis.

The TianGen Rapid DNA Extraction Kit can extract DNA from soybean powder, young shoots, or leaves in approximately 10 min. The entire extraction process does not include protein removal, RNA elimination, or the removal of other secondary metabolites and does not require organic solvent extraction or ethanol precipitation, making it simple and quick. A 5 mg soybean sample was placed in a 1.5 mL centrifuge tube, and 100 μL of Buffer B1 was added. The sample was ground repeatedly with a pestle for 30 s. After grinding, 100 μL of Buffer B2 was added, mixed by shaking, and then centrifuged at 12,000 rpm for 10 min. The oil film on the surface of the supernatant was removed, and the remaining liquid was used as the template.

### 4.3. Cas12a Expression and Purification

The LbCas12a sequence, derived from *Leptotrichia buccalis*, was cloned into the pET-28a plasmid with a C-terminal His-tag to form the expression vector (LbCas12a-pET28a). The plasmid was then transformed into Escherichia coli BL21 cells, which were cultured overnight in 100 mL of LB broth at 37 °C. The cultured cells were then transferred to 900 mL of fresh LB broth and incubated at 37 °C until the optical density at 600 nm (OD600) reached 0.8. Protein expression was induced by adding 1 mM isopropyl β-D-1-thiogalactopyranoside (IPTG), followed by incubation at 16 °C for 16 h. The cells were harvested by centrifugation and resuspended in 50 mL of lysis buffer [50 mM Tris-HCl (pH 8.0), 1.5 mM NaCl, 1 mM DTT, and 5% (*v*/*v*) glycerol]. The cells were lysed by sonication, and the lysate was centrifuged at 12,000 rpm for 30 min to collect the supernatant. Ni-NTA agarose was added, and the mixture was incubated on ice for 4 h. The bound proteins were washed with wash buffer (lysis buffer supplemented with 30 mM imidazole) and eluted using elution buffer (lysis buffer supplemented with 600 mM imidazole). The collected proteins were dialyzed in storage buffer [20 mM Tris-HCl (pH 8.0), 600 mM NaCl, 1 mM DTT, 0.2 mM EDTA, and 15% (*v*/*v*) glycerol] and stored at −80 °C for further use [29].

### 4.4. Synthesis of RPA Specific Primers and Probes

We used SnapGene (https://www.snapgene.com, accessed on 22 December 2024, Dotmatics, Boston, MA, USA) to design specific RPA primers and probes based on the transformation-specific sequence of ‘Zhonghuang 6106’ soybean. First, the target region was identified. While RPA can amplify fragments up to 1.5 kb, it is more suitable for amplicons between 100 and 200 bp. Next, multiple primers of 30–35 bases in length were designed on both sides of the target region for subsequent selection. The probe is recommended to be 46–52 bases in length and should include an abasic nucleotide to allow specific cleavage by nuclease [10]. The sequences of the primers and probes are listed in Supplementary Table S2.

### 4.5. Transcription of crRNA

The preparation of crRNA was divided into three steps. First, the transcription template required for crRNA preparation was amplified by PCR, and the primer sequences and PCR programs are listed in Supplementary Tables S3 and S4. Subsequently, the T7 High-Efficiency RNA Synthesis Kit was used for overnight transcription at 37 °C. Finally, transcription products were purified by isopropanol precipitation, and the concentration was measured using Nano-Drop 1000, aliquoted, and stored at −80 °C.

### 4.6. Amplification Methods

The PCR reaction is performed using a VeritiTM Fast PCR machine (Applied Biosystems, Thermo Fisher Scientific, USA) with a final reaction volume of 50 μL. The final reaction system contained 25 ng of DNA template, 25 μL of 2× Phanta Max Master Mix, 20 pmol each of forward and reverse primers, and 20 μL of water. The amplification steps are as follows: pre-denaturation at 95 °C for 5 min, 35 cycles of denaturation at 95 °C for 30 s, annealing at 60 °C for 30 s, extension at 72 °C for 15 s, and a final extension at 72 °C for 7 min. The RPA reaction was carried out in a metal bath with a final reaction volume of 50 μL, prepared as follows: 29.5 μL of buffer, 11.2 μL of water, 24 pmol each of forward and reverse primers, 50 ng of DNA template, and 2.5 μL of Mg^2+^ activator. The mixture was incubated at 39 °C for 20 min. Afterward, 10 μL of the RPA product was added to a 10 μL CRISPR reaction system, which consisted of 0.7 μL DEPC, 3 μL NEBTM r3 Buffer, 6.96 pmol of Cas12a protein, 54.35 pmol of crRNA, and 2 pmol of a reporter, making up a total of 20 μL. The entire system was incubated at 37 °C for 30 min. After incubation, 5 μL of the reaction product was diluted with 45 μL of ddH_2_O, mixed thoroughly, and applied to a lateral flow dipstick. The result was observed after 10 min.

### 4.7. Optimization of Reaction Conditions

To improve the RPA-CRISPR reaction system, several conditions of the RPA reaction system were optimized, including primer selection, temperature (33 °C, 35 °C, 37 °C, 39 °C, 41 °C, and 43 °C) and reaction time (10 min, 20 min, 30 min, and 40 min). In addition, the conditions of the CRISPR reaction were optimized, including temperature (33 °C, 35 °C, 37 °C, 39 °C, 41 °C, and 43 °C), reaction time (10 min, 20 min, 30 min, 40 min, 50 min, and 60 min), the reporter length (7 nt, 11 nt, and 15 nt), reporter dosage (0 nM, 10 nM, 20 nM, 30 nM, 40 nM, 50 nM, 60 nM, 70 nM, 80 nM, 90 nM, and 100 nM), Cas12a protein dosage (0 μg, 0.5 μg, 1 μg, 1.5 μg, and 2 μg), and crRNA dosage (0 ng, 250 ng, 500 ng, 750 ng, and 1000 ng).

### 4.8. Specificity Test

Several templates were used to verify the specificity of the RPA-CRISPR method, including mixed samples of GM maize, GM rice, GM cotton, GM rapeseed, and GM soybean. Their compositions are shown in Table 1. ‘Zhonghuang 6106’ was used as a positive control, while ddH_2_O served as a negative control.

### 4.9. LOD Test

The genomic template of ‘Zhonghuang 6106’ was serially diluted to concentrations of 20,000 copies/μL, 2000 copies/μL, 200 copies/μL, 20 copies/μL, 10 copies/μL, and 1 copy/μL. The limit of detection for the method was validated using both lateral flow dipstick and real-time fluorescence detection.

### 4.10. Application of the RPA-CRISPR Method in the Detection of Genetically Modified Soybean ‘Zhonghuang 6106’

To evaluate the accuracy of the RPA-CRISPR method in actual detection, we extracted 9 samples of ‘Zhonghuang 6106’ and 4 samples of ‘Zhonghuang 10’ randomly distributed in 14 1.5 mL centrifuge tubes and soaked overnight in water. The seed coats were removed, and a small number of the young shoots were used to extract DNA templates using the DNA rapid extraction kit. Known DNA samples of ‘Zhonghuang 6106’ and ‘Zhonghuang 10’ DNA were used as positive and negative controls, respectively. The Ct values of the 14 samples were determined using the QuantStudioTM 3 Real-Time PCR instrument (Applied Biosystems, Thermo Fisher Scientific, USA) as the basis for judgment. The qPCR reaction system included 12.5 μL premix Ex Taq (probe qPCR), 7.5 μL H_2_O, 10 μM forward and reverse primers each 1 μL, 2 μL DNA template, 0.5 μL 10 μM FAM-labeled probe, and 0.5 μL ROX Reference Dye Ⅱ, totaling 25 μL. The primer and probe sequences are listed in Supplementary Table S5, with sequencing referring to the ‘Detection of genetically modified plants and derived products event-specific PCR methods for herbicide-tolerant soybean zhonghuang6106’ (draft).

## Figures and Tables

**Table 1:** Composition information of transgenic mixture samples.

Mixed Sample	Composition
GM maize	Bt11, Bt176, MON810, MON863, GA21, NK603, T25, TC1507, MON89034, MON88017, 59122, MIR604, 3272, MON87460, DAS40278-9, 4114, MON87427, 5307, each at 1% content, with non-GM maize as filler
GM rice	TT51-1, KF-6, KMD-1, M12, KF-8, KF-2, G6H1, T1C-19, each at 1% content, with non-GM rice as filler
GM cotton	MON1445, MON531, MON15985, LLCOTTON25, MON88913, GHB614, COT102, each at 1% content, with non-GM cotton as filler
GM rapeseed	MS1, MS8, RF1, RF2, RF3, T45, Oxy235, Topas19/2, MON88302, 73496, each at 1% content, with non-GM rapeseed as filler
GM soybean	GTS40-3-2, MON89788, A5547-127, A2704-12, 356043, 305423, CV127, MON87701, MON87708, MON87769, MON87705, FG72, DAS81419-2, each at 1% content, with non-GM soybean as filler

## Data Availability

All authors confirm that all data and materials support their published claims and comply with field standards. These are included in this article and the Supplementary Materials.

## Supplementary Materials

The following supporting information can be downloaded at: https://www.mdpi.com/article/10.3390/ijms26010108/s1.
