# Supplementary material for: A Rapid Field-Visualization Detection Platform for Genetically Modified Soybean ‘Zhonghuang 6106’ Based on RPA-CRISPR
Source: Int J Mol Sci. 2024 Dec 26;26(1):108. doi: 10.3390/ijms26010108 (PMC11719896; doi:10.3390/ijms26010108)
Supplement: Supplementary file 1 [file ijms-26-00108-s001.zip › ijms-3370112-supplementary.pdf]

Supplementary Materials

Table S1

Table. S1 Reporter sequences used in the RPA-CRISPR method

Name	Sequence(5'FAM-3'Bio)	Length(nt)	GC Content
ssDNA1	TTTATTT	7	0%
ssDNA2	TTTTTATTTTT	11	0%
ssDNA3	TTTTTTTATTTTTTT	15	0%

Table S2

Table S2. Sequences of primers and probes used in the RPA reaction

Name	Sequence(5’-3’)
F1	TCCCGCCTTCAGTTTAAACTATCAGTGTTTGAGAC
F2	TCGTTTCCCGCCTTCAGTTTAAACTATCAGTGTTT
F3	GATTGTCGTTTCCCGCCTTCAGTTTAAACTATCAG
F4	GATCAGATTGTCGTTTCCCGCCTTCAGTTTAAACT
F5	GCTTGGATCAGATTGTCGTTTCCCGCCTTCAGTTT
F6	CTTGAGCTTGGATCAGATTGTCGTTTCCCGCCTTC
F7	AGCAGCTTGAGCTTGGATCAGATTGTCGTTTCCCG
F8	GCTAGAGCAGCTTGAGCTTGGATCAGATTGTCGTT
F9	CGAATGCTAGAGCAGCTTGAGCTTGGATCAGATTG
F10	AATGGCGAATGCTAGAGCAGCTTGAGCTTGGATCA
R1	TTTGAAAGAGCTAAATCAGAGAAAGGTGTGAAATG
R2	AACACTTTGAAAGAGCTAAATCAGAGAAAGGTGTG
R3	TGCAAAACACTTTGAAAGAGCTAAATCAGAGAAAG
R4	AATAATGCAAAACACTTTGAAAGAGCTAAATCAGA
R5	TTATTAATAATGCAAAACACTTTGAAAGAGCTAAA
R6	CCTTGTTATTAATAATGCAAAACACTTTGAAAGAG
R7	CTCACCCTTGTTATTAATAATGCAAAACACTTTGA
R8	TTGCTCTCACCCTTGTTATTAATAATGCAAAACAC
ZH6106-P	AAATCACCCACTCGTTCTGCCGCAAT(FAM)GT(THF)GT(BHQ)TA TTAAGTTGTCTAA(C3 spacer)

Table S3

Table S3. DNA template sequences of crRNA

Name	Sequence(5’-3’)
crRNA-F	GAAATTAATACGACTCACTATAGGAATTTCTACTAAGTGTAG ATAGACTGTAAATCACC
crRNA-R	AACGAGTGGGTGATTTACAGTCTATCTACACTTAGTAGAAAT TCCTATAGTGAGTCGTA

Note: These sequences represent the DNA templates for crRNA synthesis, which are used to generate the crRNA molecules for the RPA-CRISPR assay.

Table S4

Table. S4 PCR program for DNA template preparation

Temperature	Time
98℃	3min
59℃	30s
72℃	10min

Table S5

Table. S5 Specific primers/probes for GM soybean ‘ZH6106’ event

Name	Sequence(5’-3’)
ZH6106 qF	TCTAAGCGTCAATTTGTTTACATCA
ZH6106 qR	AAAGGTGTGAAATGTGAACGC
ZH6106 qP	(FAM)TTACCTAAAACATCTCAGCACATCGC(BHQ)

Figure S1

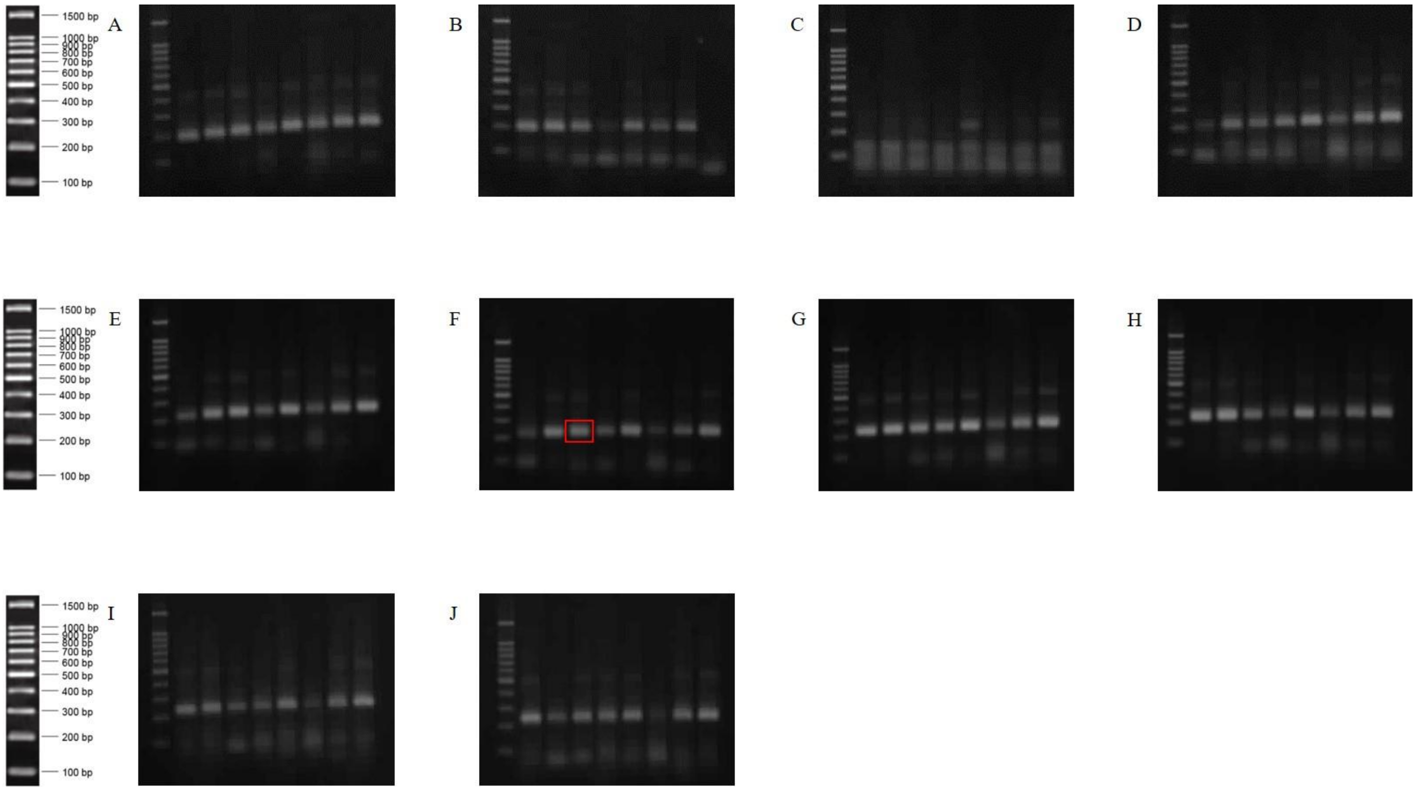

Figure S1. RPA primer screening results (A)F1,R1-R8 (B)F2,R1-R8 (C)F3,R1-R8 (D)F4,R1-R8 (E)F5,R1-R8 (F)F6,R1-R8 (G)F7,R1-R8 (H)F8,R1-R8 (I)F9,R1-R8 (J)F10,R1-R8. The final selected primer pair is F6R3, with a target fragment size of 230 bp.

Figure S2

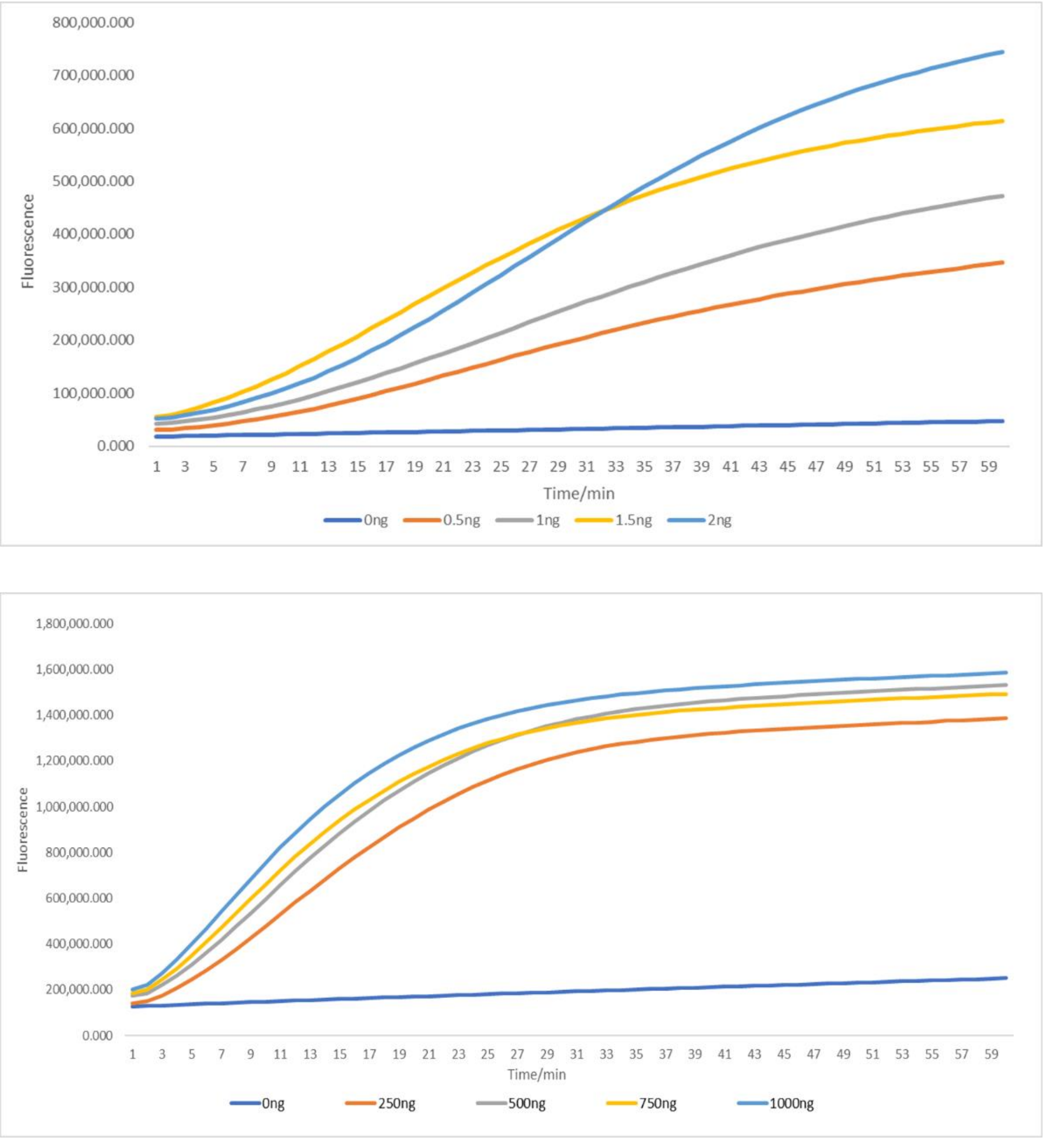

Figure S2. Real-time fluorescence results of Cas12a protein and crRNA dosage screening. (A) Cas12a protein dosage screening results. Before 32 minutes, the fluorescence intensity of the 1.5ng group was stronger than that of the 2.0ng group. Since the CRISPR reaction time is 30 minutes in the RPA-CRISPR method, 1.5ng was determined to be the optimal amount. (B) The optimal amount of crRNA. The fluorescence intensity increased with the amount of crRNA.
